# eDNA- and eRNA-Based Detection of 2-Methylisoborneol-Producing Cyanobacteria and Intracellular Synthesis Dynamics in Freshwater Ecosystem

**DOI:** 10.3390/biology14101377

**Published:** 2025-10-09

**Authors:** Keonhee Kim, Chaehong Park, Nan-Young Kim, Soon-Jin Hwnag

**Affiliations:** 1 Human and Ecocare Center, Department of Environmental Health Science, Konkuk University, Seoul 05029, Republic of Korea; passbosko@gmail.com (K.K.); celeste0@daum.net (N.-Y.K.); 2 Encounter the Ecology Co., Suwon 56192, Republic of Korea; qkrcoghd2@gmail.com

**Keywords:** 2-methylisoborneol, *mibC*, environmental DNA, environmental RNA, cyanobacteria, early warning

## Abstract

**Simple Summary:**

Off-flavors and odors in drinking water sources are often linked to the occurrence of cyanobacteria. Specific species produce the secondary metabolite 2-methylisoborneol (2-MIB), which causes a distinct earthy–musty odor that can be detected even at concentrations below the nanogram-per-liter level. Predicting the occurrence of 2-MIB remains difficult due to its sporadic production, which is influenced by both physiological state and environmental factors. Conventional monitoring approaches typically identify the compound only once accumulation is underway. In this study, we performed a longitudinal survey along the North Han River, Republic of Korea, to assess both the presence of 2-MIB-producing cyanobacteria and their active 2-MIB biosynthesis. Biweekly water samples were collected from twelve locations for over two years, and analyses included both 2-MIB concentrations and the expression levels of the biosynthetic genes. Notably, heightened expression of 2-MIB biosynthetic genes was observed approximately two to four weeks before increases in detected 2-MIB concentrations. Our results indicate that monitoring biosynthetic gene expression at the molecular level provides an effective early warning for odor episodes in drinking water supplies. Applying this approach would allow water resource managers to initiate timely interventions, ultimately protecting water quality and reducing complaints from consumers.

**Abstract:**

Taste and odor (T&O) compounds in freshwater are frequently produced by certain cyanobacteria; however, their occurrence remains difficult to predict. This study examined the temporal and spatial variations in the *mibC* gene, which encodes a critical enzyme in the biosynthesis of 2-methylisoborneol (2-MIB), by analyzing environmental DNA (eDNA) and RNA (eRNA) in the North Han River, Republic of Korea, from July 2019 to October 2021. Surface water was sampled at twelve sites and analyzed for *mibC* DNA copy number, RNA expression, cyanobacterial cell density, and 2-MIB concentration using quantitative PCR (qPCR), microscopy, and gas chromatography–mass spectrometry (GC–MS). The *mibC* gene was present throughout the year, exhibiting peaks from late summer to early winter; higher concentrations typically initiated upstream and subsequently moved downstream. RNA expression was elevated from summer to autumn, rapidly declined following heavy rainfall, and reliably preceded increases in 2-MIB concentrations by 2–4 weeks. RNA levels were strongly correlated with 2-MIB concentrations (r = 0.879, *p* < 0.001) but showed only a moderate association with *Pseudanabaena* cell density, whereas DNA demonstrated weaker correlations. More than 95% of total 2-MIB was dissolved, limiting the ability to directly estimate concentrations from eRNA data alone. The results indicate that eRNA monitoring is an effective early warning tool for T&O events. In addition, combining eDNA and eRNA analyses enables a more accurate evaluation of T&O-producing cyanobacteria, presenting practical benefits for proactive management of drinking water.

## 1. Introduction

Traditional methods for identifying sources of odor-producing compounds and the associated cyanobacteria have mainly depended on direct chemical measurements and species identification by microscopy. Along with 2-MIB, geosmin is another representative taste-and-odor compound produced by cyanobacteria [1,2]. Both compounds are responsible for the characteristic earthy–musty odors that frequently degrade the aesthetic quality of drinking water and have been reported to cause significant consumer complaints and increased treatment costs in many freshwater systems worldwide. Because they can be detected by humans at concentrations as low as a few nanograms per liter, even trace amounts of these compounds pose a challenge for water utilities. Moreover, their occurrence is often episodic and influenced by environmental factors such as temperature, nutrient availability, and hydrological conditions, which makes prediction and management particularly difficult. Generally, gas chromatography (GC) is generally applied to quantify odor compounds in environmental samples, and subsequent statistical analyses attempt to link the concentrations to likely causative cyanobacterial taxa [1,2,3,4]. However, these statistical approaches have intrinsic limitations in accurately determining the actual causative species or forecasting their appearance. Moreover, cyanobacteria’s potential to produce odor compounds differs not only between species but also among strains; some strains can generate high levels under changing environmental parameters [5,6,7,8].

The biosynthetic capacity for odor compounds in cyanobacteria is governed by the presence and nature of specific genes [9,10,11,12]. Variation in production ability occurs at both the species and strain levels; consequently, a given species may comprise both producer and non-producer strains [13,14]. However, the mere presence of biosynthetic genes does not guarantee active compound synthesis, as gene expression levels can fluctuate significantly in response to environmental parameters [15,16,17,18,19]. Because cell morphology alone cannot reveal the presence of biosynthetic genes, molecular ecological techniques targeting these genes have been designed to address this limitation [20,21,22].

Since the early 2000s, investigations of biologically derived odor compounds have increasingly utilized real-time quantitative PCR (qPCR) to detect and quantify odor-producing cyanobacteria. Environmental DNA (eDNA) analysis has also become an important complementary method, enabling the detection of both intracellular and extracellular DNA without the need for laborious cell isolation procedures [23,24,25,26,27,28,29,30,31]. This strategy supports tracking the spatial and temporal distributions of cyanobacteria that possess the capacity for, or are currently involved in, odor compound biosynthesis within aquatic habitats [32]. Recently, gene-specific primers for odor biosynthesis have been established for qPCR, facilitating targeted surveillance of *Geosmin*- and 2-methylisoborneol (2-MIB)-producing cyanobacteria in China and North America. Of particular note, field-deployable methodologies incorporating these primers have been implemented in China and Taiwan, allowing real-time monitoring of large-scale *Geosmin*-producing cyanobacterial blooms [27,28,33]. In Republic of Korea, primers for the quantitative detection of the 2-MIB biosynthesis gene (*mibC*) have been designed and utilized to monitor odor biosynthesis gene abundance in cyanobacteria [34]. Nevertheless, these investigations have predominantly focused on eDNA, and research employing environmental RNA (eRNA) approaches to evaluate cyanobacterial physiological activity remains scarce.

Environmental RNA, which is transcribed from DNA as messenger RNA, acts as a direct molecular indicator of gene expression [35]. While eDNA can confirm the presence of a species, eRNA offers additional information regarding whether specific genes are currently being expressed—and thus functionally active—which strengthens the reliability of ecological interpretation [36,37,38]. This distinction is especially significant for secondary metabolites such as odor compounds, for which the exact timing and environmental context of gene expression are more relevant than simply detecting the gene’s existence. Therefore, eRNA-based approaches allow for pinpointing when and where odor compounds are actively synthesized in aquatic systems [39,40]. Additionally, due to its rapid degradation by intracellular enzymes, eRNA reflects only recent biological activity, thereby serving as a dynamic marker of ongoing metabolic processes. Integrating eRNA with eDNA analysis facilitates a comprehensive ecological evaluation, enabling distinction between organismal presence and metabolic activity [32,41]. However, studies that utilize eRNA to assess the metabolic activity of odor-producing cyanobacteria remain limited, both within Republic of Korea and globally.

In this investigation, we targeted the *mibC* gene, which encodes the enzyme critical for 2-MIB biosynthesis in cyanobacteria, using both eDNA and eRNA extracted from freshwater samples. The aims were to (i) characterize spatiotemporal variation in cyanobacteria capable of producing 2-MIB, (ii) monitor changes over time in 2-MIB biosynthetic activity, and (iii) clarify the interconnections among genetic material, noxious cyanobacteria, and odor compound dynamics within aquatic ecosystems.

## 2. Materials and Methods

### 2.1. Study Sites and Sample Collection

**Figure 1:**
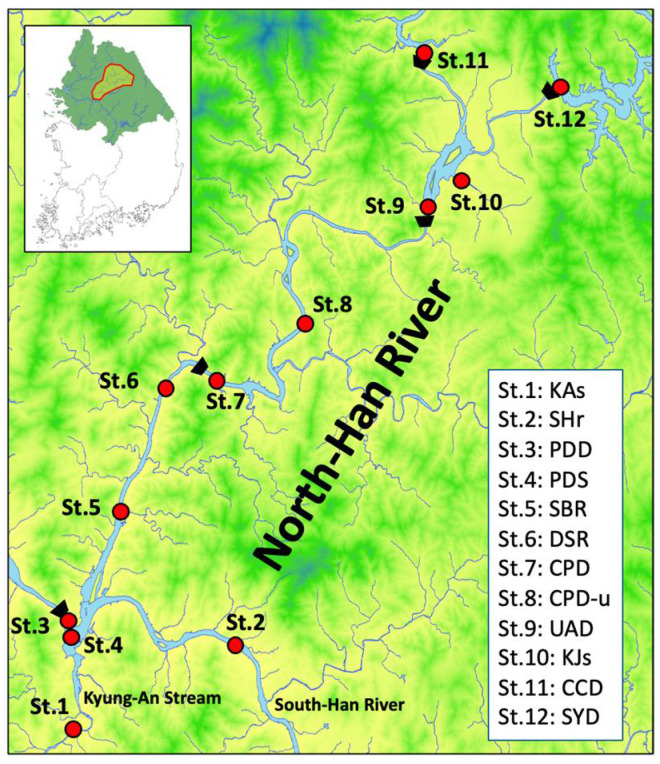
Map of the North Han river basin showing sampling locations. Twelve sampling stations (St.1–St.12) are denoted by red circles. Station codes: St.1, KAs; St.2, SHr; St.3, PDD; St.4, PDS; St.5, SBR; St.6, DSR; St.7, CPD; St.8, CPD-u; St.9, UAD; St.10, KJs; St.11, CCD; St.12, SYD. The inset illustrates the broader geographic position of the study region within Republic of Korea. Detailed information on all station codes (St.1–St.12), including abbreviations and geographic coordinates, is provided in Supplementary Table S1.

Monthly sampling was conducted at twelve designated locations in the North Han River basin between July 2019 and October 2021 (n = 28; Figure 1). All sites were targeted during each monthly sampling; however, extreme weather conditions such as flooding or ice cover occasionally prevented collection at some locations. Surface water (~30 cm depth) was collected at each site with a Van Dorn water sampler (Geo Scientific Ltd., Vancouver, Canada) before being transferred into 4 L polyethylene bottles and 100 mL glass vials. All samples were maintained in the dark and chilled during transport to the laboratory, and delivered within 24 h. In the laboratory, water samples were allocated for specific analyses as follows: (i) 1 L was fixed with 20 mL of Lugol solution for cyanobacterial cell counting, emphasizing quantification of *Pseudanabaena*; (ii) approximately 1.5 L was reserved for the assessment of the 2-methylisoborneol (2-MIB) biosynthesis gene (*mibC*); and (iii) 300 mL was placed in glass vials for in situ measurement of 2-MIB concentrations.

Water samples were filtered through GF/F glass fiber filters (pore size 0.7 µm, diameter 45 mm; Whatman, Maidstone, UK) and polycarbonate membrane filters (pore size 1.0 µm, diameter 45 mm; Whatman, Maidstone. UK) to concentrate cyanobacterial cells. eDNA and eRNA were extracted from the material retained on the filters to evaluate the spatiotemporal distribution and expression of the *mibC* gene. Filtration continued until filter clogging occurred, after which the total filtered volume was recorded for each sample. Filters containing eDNA and eRNA were transferred into Salivette tubes and 5 mL tubes, respectively, and then stored at −20 °C until nucleic acid extraction (Figure S1).

### 2.2. eDNA and eRNA Extraction

In the laboratory, eDNA and eRNA retained on the GF/F and polycarbonate filters were extracted using distinct protocols. For eDNA extraction, cells accrued on GF/F filters were physically lysed by repeated freeze–thaw cycles, followed by enzymatic digestion with Proteinase K and lysis buffer at 56 °C for 30 min. The resulting lysate was purified employing the DNeasy Blood and Tissue Kit (Qiagen Co., Hilden, Germany), adhering to the manufacturer’s protocol for eDNA extraction [42]. eDNA was eluted using 100 µL of elution buffer.

For eRNA extraction, 1 mL of Trizol-based RiboEX reagent (GeneAll Co., Daejeon, Republic of Korea) was introduced into a 5 mL conical tube containing the polycarbonate filter. The tubes were rotated at room temperature to ensure thorough contact between the reagent and the filter surface, which promotes the release of RNA from the retained cells. RNA was then purified utilizing the Hybrid-R^TM^ RNA purification kit (GeneAll Co., Daejeon, Republic of Korea), which includes an on-column DNase treatment step to remove residual genomic DNA, and subsequently eluted in 70 µL of elution buffer.

The concentrations of extracted eDNA and eRNA were assessed using a Qubit 4 Fluorometer (Thermo Fisher Scientific, Waltham, MA, USA), and samples were maintained at −80 °C until further analysis.

### 2.3. Quantitative Analysis of eDNA and eRNA

Quantitative PCR (qPCR) was employed to detect *mibC* genes in environmental DNA and RNA, quantify DNA copy numbers, and analyze gene expression, using a Rotor-Gene instrument (Takara, Kusatsu-shi, Japan). The primers utilized for *mibC* amplification were mibC 300F (5′-TGT TAC GCC ACC TTC TCT ATG TT-3′) and mibC 300R (5′-CAA TCT GTA GCA CCA TGT TGA-3′), as described previously [34].

For eDNA analysis, each 20 µL reaction mixture consisted of 10 µL of 2× Real-Time PCR Master Mix with SFCgreen^®^ I (BioFACT Co., Daejeon, Republic of Korea), 1 µL each of the forward and reverse primers (10 pmol/µL), 5 µL of nuclease-free water, and 3 µL of template DNA. The thermal cycling protocol included an initial denaturation at 95 °C for 15 min, then 35 cycles of 95 °C for 5 s, 62 °C for 10 s, and 72 °C for 15 s, with fluorescence detected at the 72 °C step of each cycle.

For *mibC* gene expression analysis, complementary DNA (cDNA) was synthesized from DNase-treated RNA and quantified using OneStep qRT-PCR Master Mix with SFCgreen^®^ I (BioFACT Co., Daejeon, Republic of Korea), according to the manufacturer’s protocol. All qPCR reactions were run in triplicate, and no-template negative controls were included in each assay. The reaction mixtures were prepared identically to those for eDNA analysis, except that RNase-free water replaced nuclease-free water. The thermal program involved cDNA synthesis at 95 °C for 30 min, followed by qPCR initiation at 95 °C for 15 min, and then 40 cycles of 95 °C for 5 s, 62 °C for 10 s, and 72 °C for 15 s, with fluorescence detection at the 72 °C step.

(1) $$N_{pc}=\frac{C_{T}\times A_{N}}{S_{A}\times {MW}_{bp}\times 10^{6}}$$

Gene copy numbers (*N_pc_*) were estimated from Ct values (threshold cycle in qPCR) using a standard curve according to Equation (1) [43], in which *A_N_* is Avogadro’s number, *MW_bp_* denotes the average molecular weight of a base pair, *S_A_* represents the genome size of *Pseudanabaena*, and *C_T_* refers to the target gene copy number derived from the standard curve. A genome size of 4.8 × 10^6^ bp was assumed for *Pseudanabaena* sp. Chao 1811 strain [44]. The standard curve was established using *Pseudanabaena galeata* NIES-512 as the calibration reference, with copy numbers determined via cloning-based methods [43,45]. The amplification efficiency of the standard curve was 92.3%, with an R^2^ value of 0.996, indicating high reproducibility of the assay. This strain was chosen because Pseudanabaena spp. were dominant in the study region, and the sequence information of NIES-512 was included in the design of the *mibC* primers, making it the most suitable reference for this study.

### 2.4. Cyanobacterial Cell Density and 2-MIB Analysis

Samples preserved with Lugol’s solution were concentrated by settling naturally for a minimum of 72 h. A 1 mL subsample was transferred into a Sedgwick–Rafter counting chamber, allowed to settle for at least 10 min, and observed under a light microscope. The morphological characteristics of the identified taxa were documented using a camera mounted on the microscope and compared with published taxonomic descriptions [46,47,48].

For 2-MIB analysis, the headspace solid-phase microextraction (HS-SPME) technique was utilized [49]. Cell suspensions disrupted by ultrasonication were directly injected into a GC/MS system (450-GC, 320-MS; Bruker, Billerica, MA, USA) and thermally desorbed at 270 °C for 4 min. Target analyte peaks were identified based on retention time and quantified by integrating the peak area.

### 2.5. Statistical Analysis

Heatmaps depicting *mibC* DNA and RNA copy numbers (copies/mL) were generated using R (v.3.6.0) and R Studio (v.1.1.463) with the ggplot2 package (v.3.1.1) [50]. Final visualizations were further refined with DataGraph (v.4.4; Visual Data Tools Inc., Chapel Hill, NC, USA).

To examine the associations between 2-MIB concentration and *mibC* RNA expression, six datasets were randomly selected using the INDIRECT and RAND functions in Excel. Regression equations were established for each dataset, and the sampling procedure was repeated more than 50 times. Only regressions with correlation coefficients (r) ≥ 0.8 were included in subsequent analyses, to emphasize the most robust predictive relationships and to reduce potential noise from weak or inconsistent regressions. For these selected regressions, model adequacy was examined using the coefficient of determination (R^2^) and F-statistics, and significance was determined by *p*-values (*p* < 0.05 was regarded as statistically significant).

To assess the relationships among cyanobacterial cell density, 2-MIB concentration, *mibC* DNA copy number, and *mibC* RNA expression, Pearson correlation analyses were performed using JASP (v.0.19.3), and the findings were displayed as scatter plots. To further investigate the impact of the August 2020 flood, two-way ANOVA was conducted with period (pre- vs. post-flood) and site as fixed factors on log_10_-transformed *mibC* gene and transcript abundances. Additionally, linear mixed-effects models incorporating sites as a random intercept were fitted to verify effects related to flood period. Holm’s method was applied to adjust post hoc comparisons.

## 3. Results

### 3.1. Spatiotemporal Variation in mibC Gene DNA Copy Numbers

**Figure 2:**
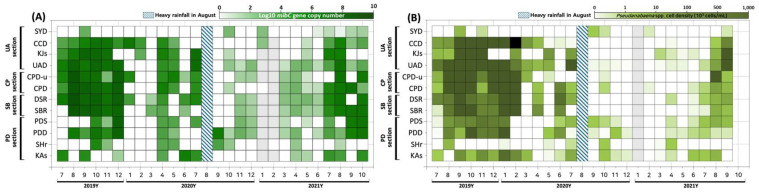
Temporal and spatial variation in *mibC* DNA copy numbers in the North Han River basin from July 2019 to October 2021. (**A**) Temporal variation in *mibC* gene DNA copy numbers. (**B**) Spatial distribution of *Pseudanabaena* spp. cell density. The color scale depicts log_10_-transformed *mibC* gene copy numbers (copies/mL) and *Pseudanabaena* spp. cell density (10^3^ cells/mL). The hatched vertical bar denotes the timing of the August 2020 heavy rainfall event.

Between July 2019 and October 2021, the *mibC* gene, which is integral to 2-MIB biosynthesis, was regularly identified at most sampling sites throughout the North Han River basin, with maximum abundance typically recorded from late summer (August) to early winter (December) (Figure 2). A marked alteration in spatial and temporal distribution was observed before and after the significant rainfall event in August 2020.

Prior to the major rainfall, the highest *mibC* concentrations were mainly detected in the upstream regions. During July–August 2019, 10^7^–10^8^ copies/mL were measured in the CP section (CPD, CPD-u) and the UA section (UAD, KJs, CCD, SYD). At KJs, *mibC* gene copy numbers rose to 3.5 × 10^4^ copies/mL in September 2019, whereas at downstream site USD, concentrations increased by more than 100-fold to 2.2 × 10^7^ copies/mL in October. Elevated concentrations subsequently appeared downstream at PDD and PDS, with an average of 3.3 × 10^5^ copies/mL. Starting in November, *mibC* became undetectable until isolated reappearance from January–February 2020 at certain locations, including CCD and CPD-u (10^4^–10^5^ copies/mL in January), and CCD and KJ (10^3^–10^4^ copies/mL in February), even as water temperatures dropped below zero.

Between April and July 2020, CPD and UAD displayed concentrations of 10^4^–10^6^ copies/mL, with the UA section peaking before the rainfall event (UAD: 8.0 × 10^6^ copies/mL; KJs: 5.2 × 10^6^ copies/mL). Immediately after the August rainfall, *mibC* was only detected at SHr in September, with no detection at other locations. The gene reappeared from October in SYD, UAD, CCD, and PDD, but at substantially lower levels (14 to 612 copies/mL). From September to December 2020, detected concentrations were between 9–10^3^ copies/mL, which was around 10^5^ copies/mL lower than concentrations from the same months in 2019.

In March 2021, shortly after ice melted, *mibC* was detected from PDD to KJs, with concentrations ranging from 24 to 307 copies/mL, and the highest reading occurred between DSR and CPD-u. Nonetheless, these concentrations were >10^4^ copies/mL lower than those recorded in April 2020, and high-concentration areas had shifted below UAD. In June–July 2021, marked increases were noted at most sampling points (10^3^–10^5^ copies/mL); SBR–CPD registered 3.7 × 10^4^–7.4 × 10^4^ copies/mL, while CPD-u continued to show negative results. KJs measured 1.1 × 10^5^ copies/mL, representing the highest value detected in the UA section. In August 2021, concentrations reached a mean of 2.3 × 10^6^ copies/mL between SBR and UAD, with an increase in CPD-u–UAD from 1.5 × 10^6^ to 7.9 × 10^6^ copies/mL. In September–October, both PDD and PDS in the downstream area showed elevated concentrations compared to 2019, but overall abundance decreased by 2–4-fold.

In summary, elevated *mibC* DNA copy numbers initially appeared in upstream sections and later shifted to downstream areas. Even after significant rainfall, *mibC* was detected at most sites, but regions of high concentration became more localized and DNA copy numbers fell by more than two-fold. Two-way ANOVA suggested a borderline effect of flood period (F_1_,_281_ = 3.85, *p* = 0.051), while a mixed-effects model demonstrated a significant decline in gene copy number after the flooding event (β = −0.522 ± 0.264, *p* = 0.047), indicating an approximately 70% reduction in abundance (to 0.30-fold compared to pre-flood levels).

### 3.2. Spatiotemporal Variation in mibC Gene Expression (RNA)

**Figure 3:**
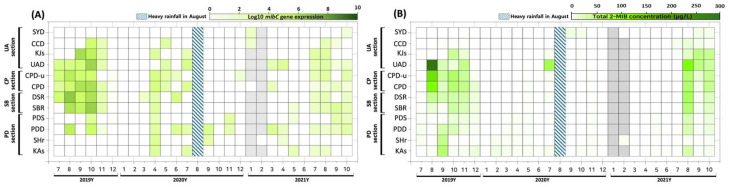
Comparison of *mibC* DNA and RNA expression in the North Han River Basin from July 2019 to October 2021. (**A**) Spatiotemporal variation in *mibC* gene expression. (**B**) Site-specific differences in total 2-MIB concentration. Colors represent log_10_-transformed *mibC* RNA copy numbers (copies/mL) and total 2-MIB concentrations (µg/L). The hatched vertical bar indicates the period of the August 2020 heavy rainfall event.

In the North Han River, *mibC* expression peaked during summer to fall and largely paralleled the trends observed in DNA copy numbers (Figure 3). In July 2019, expression (1.3 × 10^3^–4.2 × 10^4^ copies/mL) was confined to sites between UAD and DSR, whereas expression was not detected at CCD and KJs. From August to October 2019, expression sharply increased, with an average of 1.0 × 10^6^ copies/mL between SBR and KJs—representing an approximately 100-fold elevation from July (1.1 × 10^4^ copies/mL). The maximum 2019 expression occurred at DSR (6.3 × 10^6^ copies/mL). Consistently high expression was found throughout SBR–KJs, with CCD also reaching 4.2 × 10^4^ copies/mL in October. By November, expression levels had decreased by over 1000-fold at all sites and became nearly undetectable by December (1–3 copies/mL).

During the ice-covered months of January–February 2020, *mibC* DNA (7.9 × 10^3^–5.3 × 10^4^ copies/mL) was observed at DSR and CPD-u, but no RNA expression was detected. Expression resumed in March 2020 (DSR: 45 copies/mL), with levels increasing more than 100-fold in April (mean: 2.9 × 10^3^ copies/mL, range: 2.2 × 10^2^–1.1 × 10^4^ copies/mL) at most sites; both CPD and CPD-u exceeded 7.1 × 10^3^ copies/mL. The SHr site recorded 2.3 × 10^3^ copies/mL, which was more than double the values downstream. After April, expression was found at fewer locations but persisted through July, ranging from 7.4 × 10^2^ to 8.8 × 10^3^ copies/mL. In the aftermath of the August rainfall, expression was limited to PDD (2.0 × 10^3^ copies/mL) and SHr (458 copies/mL) in September, and declined rapidly between October and December, with only trace amounts observed at PDD, PDS, and CPD-u.

In January 2021, expression remained below 100 copies/mL at CCD and SYD. During the spring (March–May), elevated expression was observed in slow-flowing zones, with a peak at PDD (2.4 × 10^3^ copies/mL in March). Expression was not detected in June, but a rapid increase was observed in July, with high values recorded between CPD-u and KJs, where elevated expression continued into August at UAD, KJs, and CCD (UAD: 3.8 × 10^3^ copies/mL). After August, several downstream sites showed increased expression, with SBR reaching its peak in September (1.4 × 10^3^ copies/mL) followed by a roughly tenfold decrease in October.

Overall, *mibC* expression predominantly occurred from July through October, with several occurrences in October–November where DNA was detectable but RNA was absent. Expression was low during winter and spring, and following periods of heavy rainfall, expression levels declined by more than an order of magnitude. Flooding exerted a statistically significant effect, supported by both ANOVA (F_1_,_280_ = 11.32, *p* = 0.0009) and mixed-effects analysis (β = −0.604 ± 0.181, *p* = 0.001). Post-flood expression was approximately 25% of pre-flood values (0.25-fold).

Temporal comparisons demonstrated that peaks in *mibC* RNA expression consistently occurred about one month before increases in 2-MIB concentrations, indicating a lead–lag dynamic between gene transcription and metabolite accumulation. Detected 2-MIB concentrations in water were mostly associated with *mibC* RNA expression above ~10–100 copies/mL; in contrast, no RNA expression corresponded to non-detectable 2-MIB. Peak 2-MIB levels aligned with higher RNA abundances (10^3^–10^6^ copies/mL).

### 3.3. Relationship Between eRNA Expression and 2-MIB Concentration

**Figure 4:**
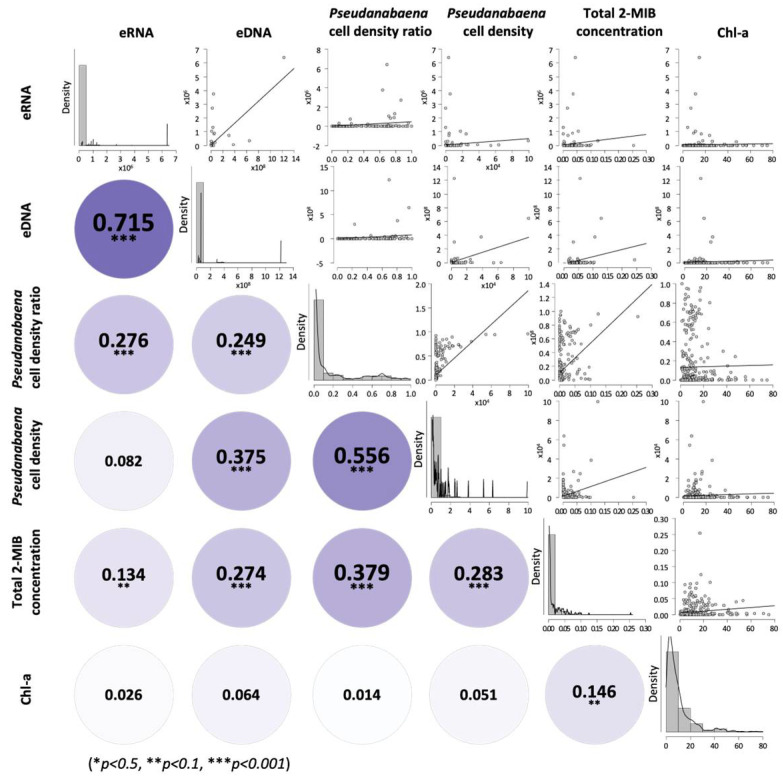
Correlation graph of *mibC* gene copies, *mibC* gene expression, cell density of the *Pseudanabaena* spp. and 2-MIB concentrations (µg/L) in water samples from study sites. Spearman correlation coefficients (ρ) between two variables are presented in boxes on the top right, corresponding bivariate scatter plots are shown on the bottom left, and histograms for each variable appear on the diagonal. Circle color intensity represents the correlation coefficient (r); darker shading indicates stronger correlations. The significance of the Spearman correlation is indicated as * *p* < 0.5, ** *p* < 0.1, *** *p* < 0.001.

**Figure 5:**
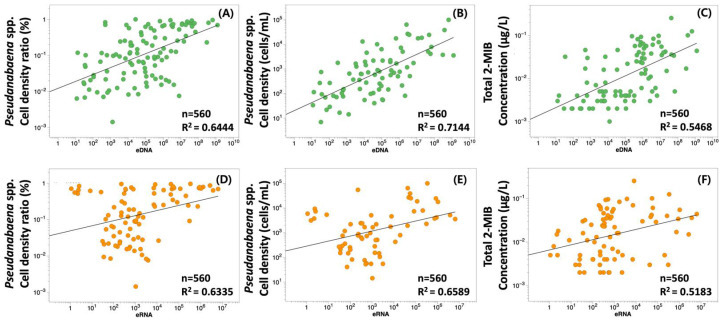
Relationships between *mibC* DNA copy numbers (top row) or *mibC* RNA expression (bottom row) and (**A**,**D**) relative abundance of *Pseudanabaena* spp., (**B**,**E**) cell density of *Pseudanabaena* spp., and (**C**,**F**) total 2-MIB concentration in the North Han River Basin. Green dots indicate DNA-based data; orange dots correspond to RNA-based data. Pearson correlation coefficients (r) and associated *p*-values are provided for each relationship.

*mibC* DNA copy number and RNA transcript abundance were associated with *Pseudanabaena* cell density, relative abundance, and 2-MIB concentrations in water (Figure 4). *mibC* DNA was significantly correlated with *Pseudanabaena* cell density (r = 0.249, *p* < 0.001), relative abundance (r = 0.276, *p* < 0.001), and 2-MIB concentration (r = 0.146, *p* < 0.01). When analysis was limited to samples exhibiting detectable DNA and RNA (Figure 5), cell density was correlated with DNA copy number (r = 0.274, *p* < 0.001) and with relative abundance (r = 0.379, *p* < 0.001). 2-MIB concentration was also correlated with DNA copy number (r = 0.283, *p* < 0.001), though this relationship was less robust (Figure 5A–C).

*mibC* RNA expression showed a statistically significant correlation with cell density (r = 0.556, *p* < 0.001) but was not correlated with relative abundance (r = 0.082, n.s.; Figure 5D,E). Among 24 samples where both RNA expression and 2-MIB were detected, 2-MIB concentrations ranged from 50 to 2671 µg/L and had a strong correlation with RNA expression (log_10_ (y) = 0.1184 × log_10_ (x) − 2.182, r = 0.879, *p* < 0.001; Figure 5F).

**Figure 6:**
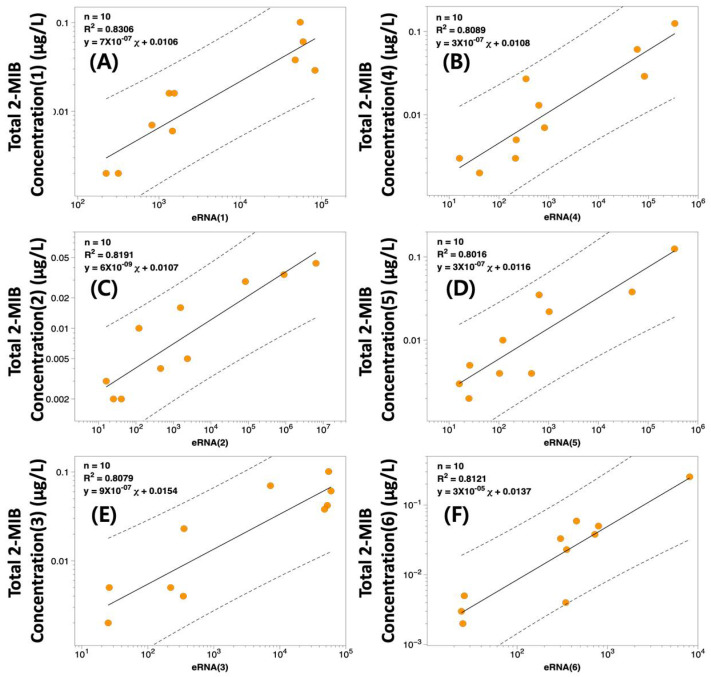
Relationship between *mibC* RNA expression and 2-MIB concentration based on randomly selected eight datasets from water samples with detection of both variables. Regression equations and relevant correlation coefficients from six representative subsets consistently exhibited positive linear associations. (**A**–**F**) Regression models derived from six independent subsets, each showing a significant positive correlation between mibC RNA expression and 2-MIB concentration.

**Figure 7:**
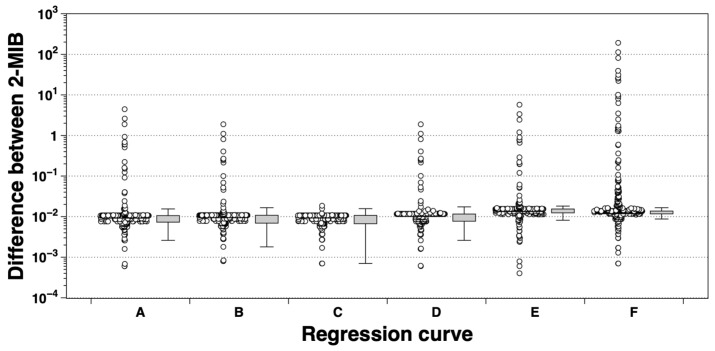
Comparison of the calculated 2-MIB concentration (cMIB) derived from the regression equations in Figure 6 and the analyzed 2-MIB concentration (aMIB) measured by GC–MS. Panels **A**–**F** correspond to the regression models presented in Figure 6. The box plots display the distribution of differences between cMIB and aMIB. the box boundaries represent the interquartile range, the whiskers indicate the minimum and maximum values, the black line denotes the median, and the white circles represent individual raw data points.

From these analyses, six regression models (derived from random subsets; Figure 6, Table S2) produced coefficients of determination (r^2^) between 0.802 and 0.831, demonstrating consistently strong linear relationships. Regression equations in log–log form and their Root Mean Square Errors (RMSE) were calculated to assess model fit. Of the six models, regression (E) most closely matched predicted and measured 2-MIB values, showing the lowest RMSE (33,081 µg/L), while the remaining models had greater prediction errors (RMSE range: 26,404–224,754 µg/L). Predicted 2-MIB concentrations (c2-MIB, estimated from *mibC* RNA expression) at times deviated from measured values (a2-MIB, actual observations), with average predictions ranging from 185 to 359 µg/L and deviations of –119 to 55 µg/L, occasionally exceeding a twofold discrepancy (Figure 7).

## 4. Discussion

### 4.1. Cyanobacterial Abundance and 2-MIB Occurrence

Numerous previous studies have demonstrated that cyanobacterial cell density does not always show a consistently strong positive correlation with in situ concentrations of odor compounds, and the magnitude and statistical significance of these correlations depend on both the type of compound and the taxonomic characteristics of the producing species [51,52,53]. In the present study, both cell density (r = 0.249, *p* < 0.001) and relative abundance (r = 0.276, *p* < 0.001) of *Pseudanabaena* spp.—the dominant taxa in this system—were statistically significant, though weakly correlated (r < 0.3), with 2-MIB concentrations. This finding likely results from the presence of both 2-MIB-producing and non-producing strains coexisting within the study area. Notably, *Pseudanabaena* populations in the North Han River consist of multiple co-occurring species with varying capacities for 2-MIB production [54]. In addition, 2-MIB biosynthesis can be either retained or lost as a result of genetic variations or deletions in the *mibC* operon [55,56,57], and even among genetically identical strains, production can vary significantly due to differences in metabolic pathways or environmental adaptability [58,59,60]. During this study, certain sites showed high *Pseudanabaena* densities but low 2-MIB concentrations, and the reverse was also observed, suggesting that intraspecific variation in production potential is a significant source of uncertainty in field monitoring.

### 4.2. Predictive Value of mibC Expression

In several regression models, *mibC* eRNA expression showed a strong correlation with 2-MIB concentrations (r = 0.938–0.989). However, predicted concentrations (c2-MIB) derived from regression equations either overestimated or underestimated the observed concentrations (a2-MIB), with deviations spanning from –119 to 55 µg/L, and in certain instances surpassing a twofold difference. The Root Mean Square Error (RMSE) values for these regressions, which ranged from 26,404 to 224,754 µg/L, provide further evidence of considerable variability in predictive accuracy across different models, emphasizing the importance of calibrating models for specific hydrological and environmental scenarios [61,62]. It should also be noted that reverse transcription efficiency can introduce systematic biases, as minor inconsistencies in cDNA synthesis may be amplified during PCR [63,64,65,66]. Moreover, environmental RNA is inherently unstable and prone to degradation, which may lead to underestimation of transcript abundances in natural samples. These factors were considered when interpreting the relationship between mibC expression and 2-MIB concentrations. Interspecies differences in transcription efficiency or RNA stability may also contribute to overestimation. Environmental variables relevant to odorant production, such as nitrogen concentration and N:P ratios, significantly affect both the potential for 2-MIB production and *mibC* gene expression, thereby influencing spatial and temporal concentration patterns [67,68,69]. Furthermore, under stress conditions like temperature fluctuations, light limitation, grazing, or cell lysis, particulate (cell-bound) 2-MIB may be released into the dissolved phase [13,70], resulting in further discrepancies between intracellular particulate amounts and dissolved concentrations in the aquatic environment. These storage–release processes likely help explain the disparity observed between expression-based predictions and directly measured total concentrations. In addition, GC–MS provides direct measurements of 2-MIB within minutes, whereas PCR-based quantification requires at least one full day including nucleic acid extraction, reverse transcription, and amplification, which constrains its applicability for real-time monitoring.

### 4.3. Environmental and Methodological Influences

In this study, *mibC* eRNA expression was quantified at approximately 429 copies/mL during summer, indicating that 2-MIB-producing cyanobacteria remain metabolically active even in deeper, cooler water layers. This occurrence is likely associated with environmental factors such as thermal stratification, nutrient buildup in deeper zones, and diminished light availability. Notably, estimates derived from *mibC* eRNA expression reflect only intracellular particulate 2-MIB and do not include concentrations present in the dissolved phase. In field measurements, total 2-MIB (sum of particulate and dissolved) consistently surpassed predictions based solely on gene expression. A recent investigation that separated 2-MIB into particulate and dissolved phases at five representative sites from March to October reported that the majority of 2-MIB was in the dissolved state, with particulate fractions having only a minor presence [71]. Particulate 2-MIB was not found at Paldang Dam and Sambong-ri, and only trace levels were observed at Daeseong-ri and upstream Cheongpyeong Lake during October. These findings demonstrate that 2-MIB in the North Han River predominantly exists in the dissolved phase, making it challenging to estimate total concentrations using gene expression data alone. Beyond phase partitioning, previous studies have shown that the biosynthesis of 2-MIB is tightly linked to cellular photophysiology and carbon overflow [13,72]. Light intensity, photosynthetic activity, and cellular energy balance directly influence the rate of secondary metabolite production, with 2-MIB synthesis increasing when photosynthetic efficiency is altered by environmental stress. According to the overflow hypothesis, 2-MIB may be synthesized when excess fixed carbon cannot be fully incorporated into primary metabolism, functioning as a metabolic outlet for surplus resources. Such photophysiological and metabolic controls provide a mechanistic explanation for why *mibC* expression and measured 2-MIB concentrations may diverge under varying conditions, beyond the differences between particulate and dissolved phases. While measurements of intracellular particulate 2-MIB provide insight into active gene transcription, eRNA detection captures metabolic processes in real time rather than reflecting pooled compound quantities. Despite this, *mibC* eRNA expression reliably preceded subsequent increases in overall 2-MIB concentrations. In the majority of cases, eRNA expression was observed prior to any measurable 2-MIB, with elevated concentrations recorded in following monitoring efforts. This lag period was generally in the range of 2–4 weeks, offering a key early-warning period advantageous for reservoir management [73,74]. The consistent temporal offset further substantiates the value of eRNA monitoring as a predictive tool for impending 2-MIB events. Considering that 2-MIB can prompt taste-and-odor issues at concentrations of only a few µg/L [75,76], the use of expression-based early-warning systems could significantly enhance the management of drinking water sources.

### 4.4. Limitations and Future Perspectives

This study also provides important insights into the biology of *Pseudanabaena*. While certain strains possess the genetic capacity to synthesize 2-MIB, not all express *mibC* or are actively engaged in producing this metabolite. As a result, increases in *Pseudanabaena* cell density do not always correlate with higher levels of *mibC* RNA expression or increases in 2-MIB concentration [54]. This finding highlights the limitation of depending solely on cell density measurements at the species or genus level for accurate odor risk evaluation. In summary, the integrated eDNA–eRNA approach utilized here identifies seasonal variation among 2-MIB-producing cyanobacteria, uncovers their environmental determinants, and characterizes the distribution of 2-MIB between dissolved and particulate phases. Notably, the temporal lead observed for eRNA expression preceding 2-MIB buildup offers significant potential for early-warning systems, and this method could become an important tool for long-term evaluations of how climate and hydrological fluctuations impact cyanobacterial metabolism and odorant generation. In addition, this study focused on surface water (~30 cm), as taste-and-odor events typically originate in the upper layer where cyanobacteria are most active. While this approach was appropriate for assessing surface-driven odor problems, incorporating depth profiles in future studies could provide additional insights into vertical heterogeneity during stratified bloom events.

## 5. Conclusions

From a management standpoint, incorporating eRNA surveillance into conventional water quality monitoring frameworks could greatly improve the early identification of taste-and-odor (T&O) outbreaks. Standard programs primarily use microscopy-based cell enumeration and infrequent chemical assessments, which commonly detect 2-MIB only after consumer complaints arise due to elevated concentrations. In contrast, the use of molecular diagnostics such as *mibC* eRNA quantification would empower water utilities to forecast odor occurrences several weeks ahead, thus enabling timely deployment of mitigation strategies like selective withdrawal, aeration, or pre-oxidation treatments.

In addition, routine adoption of eDNA–eRNA assessment can be aligned with automated, high-throughput analytical workflows, supporting integration into national water quality benchmarks and regulatory protocols. With the projected increase in cyanobacterial bloom incidents due to climate change and altered hydrological conditions, the use of expression-based early-warning techniques will become crucial for safeguarding drinking water quality.

This study also demonstrates the scientific value of integrating eDNA and eRNA approaches for simultaneously capturing genetic potential and active expression, thereby providing a more holistic understanding of odor compound dynamics. From a policy perspective, embedding molecular diagnostics into regulatory monitoring frameworks could substantially strengthen proactive management of freshwater resources and ensure safer drinking water supplies under future environmental challenges.

## Data Availability

The original datasets supporting the conclusions of this article are available from the authors upon request.

## Supplementary Materials

The following supporting information can be downloaded at: https://www.mdpi.com/article/10.3390/biology14101377/s1. Table S1: Description of sampling sites (St.1–St.12), including site abbreviation and geographic coordinates (latitude and longitude); Table S2: Regression results between *mibC* RNA expression and 2-MIB concentration. Summary of six regression models (log–log form) selected from random subsets. For each iteration, the regression equation, coefficient of determination (R^2^), and Root Mean Square Error (RMSE) are reported; Figure S1: Overview of the workflow applied in this study, from sample collection and filtration to eDNA/eRNA extraction, quantitative PCR, chemical analysis of 2-MIB, and statistical evaluation.
