# Supplementary material for: eDNA- and eRNA-Based Detection of 2-Methylisoborneol-Producing Cyanobacteria and Intracellular Synthesis Dynamics in Freshwater Ecosystem
Source: Biology (Basel). 2025 Oct 9;14(10):1377. doi: 10.3390/biology14101377 (PMC12561784; doi:10.3390/biology14101377)
Supplement: Supplementary file 1 [file biology-14-01377-s001.zip › biology-3864442-supplementary/Table S1.pdf]

Table S1. Regression results between *mibC* RNA expression and 2-MIB concentration. Summary of six regression models (log–log form) selected from random subsets. For each iteration, the regression equation, coefficient of determination (R^2), and Root Mean Square Error (RMSE) are reported.

Iteration	Equation	R^2	RMSE (raw, μg/L)	RMSE (log_{10})
1	log10(y) = 1.5888·log10(x) + 6.5809	0.831	35571.95	5.499
2	log10(y) = 3.4495·log10(x) + 10.4868	0.819	224753.74	5.528
3	log10(y) = 2.0515·log10(x) + 6.8918	0.808	26403.703	5.089
4	log10(y) = 2.1717·log10(x) + 7.2912	0.809	69196.283	5.074
5	log10(y) = 2.1794·log10(x) + 6.9856	0.802	33080.675	4.702
6	log10(y) = 1.0616·log10(x) + 4.2758	0.812	1470.351	4.168
