# Supplementary material for: eDNA- and eRNA-Based Detection of 2-Methylisoborneol-Producing Cyanobacteria and Intracellular Synthesis Dynamics in Freshwater Ecosystem
Source: Biology (Basel). 2025 Oct 9;14(10):1377. doi: 10.3390/biology14101377 (PMC12561784; doi:10.3390/biology14101377)
Supplement: Supplementary file 1 [file biology-14-01377-s001.zip › biology-3864442-supplementary/Table S2.pdf]

Table S2. Description of sampling stations (St.1–St.12), including site abbreviation and geographic coor-dinates (latitude and longitude).

Site number	Code	Site description	Coordinate	
			N	E
St.1	KAs	Kyung-an stream inflow into Paldang Lake	37° 28′ 20.40′′	127° 12′ 49.77′′
St.2	SHr	South-Han River inflow into Paldang Lake	37° 31′ 08.15′′	127° 22′ 26.04′′
St.3	PDD	Near the Paldang Lake Dam outlet	37° 31′ 19.14′′	127° 17′ 02.38′′
St.4	PDS	Near the So-ne Island in Paldang Lake	37° 30′ 26.06′′	127° 17′ 46.11′′
St.5	SBR	Downstream of North-Han River	37° 35′ 16.53′′	127° 20′ 19.59′′
St.6	DSR	Downstream of North-Han River	37° 42′ 02.31′′	127° 23′ 29.01′′
St.7	CPD	Near the Cheongpyeong Lake Dam outlet	37° 43′ 30.18′′	127° 25′ 32.77′′
St.8	CPD-u	Upstream of Cheongpyeong Lake	37° 48′ 20.71′′	127° 31′ 46.26′′
St.9	UAD	Near the Uiam Lake Dam outlet	37° 50′ 20.93′′	127° 40′ 32.02′′
St.10	KJs	Kong-ji stream inflow into Uiam Lake	37° 52′ 27.58′′	127° 42′ 34.25′′
St.11	CCD	Near the Chuncheon Lake Dam outlet	37° 58′ 14.63′′	127° 40′ 13.20′′
St.12	SYD	Near the So-yang Lake Dam outlet	37° 56′ 46.58′′	127° 49′ 04.22′′
