# Supplementary figures and images for: eDNA- and eRNA-Based Detection of 2-Methylisoborneol-Producing Cyanobacteria and Intracellular Synthesis Dynamics in Freshwater Ecosystem
Source: Biology (Basel). 2025 Oct 9;14(10):1377. doi: 10.3390/biology14101377 (PMC12561784; doi:10.3390/biology14101377)

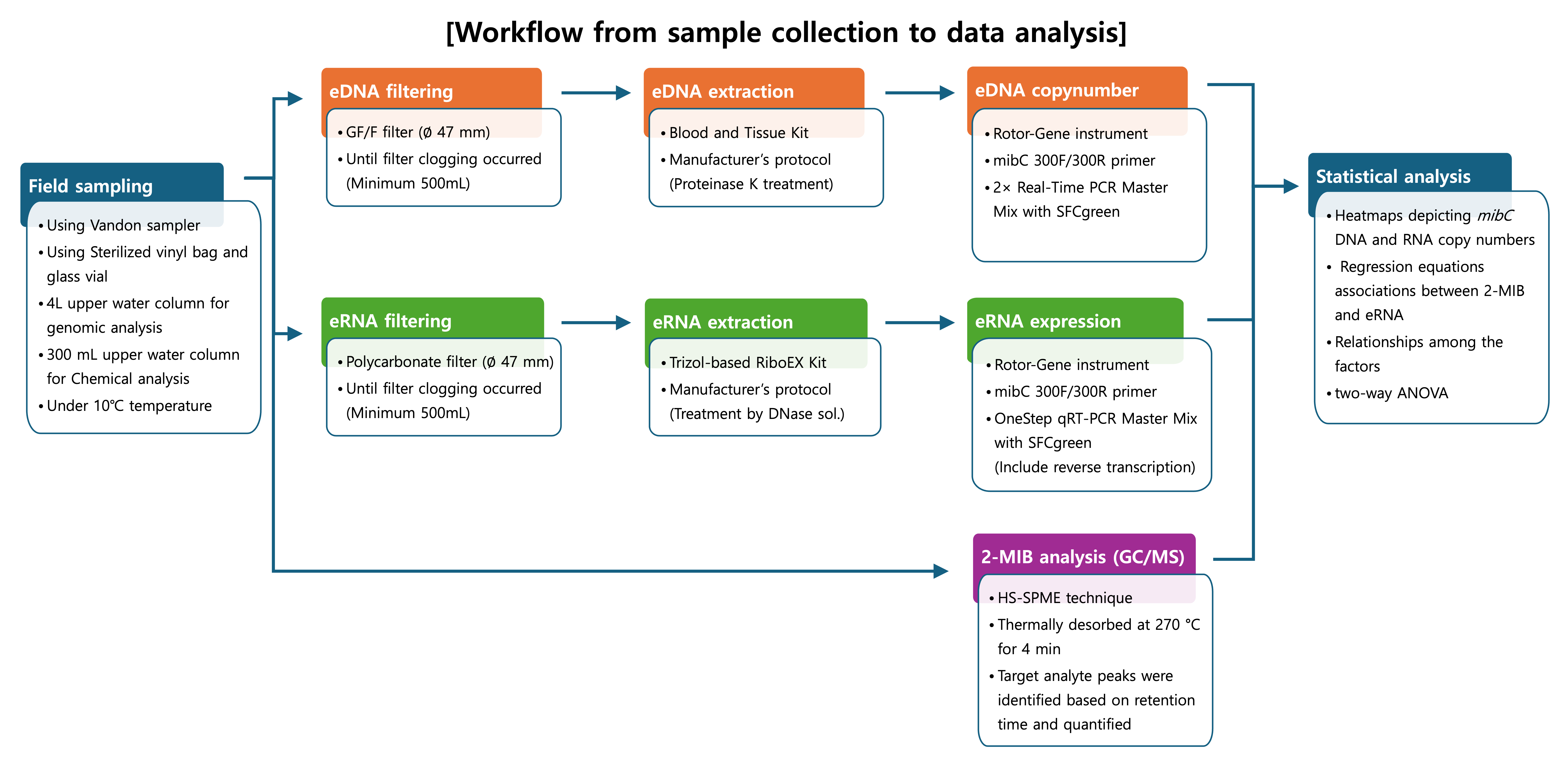

Supplement: Supplementary file 1 [file biology-14-01377-s001.zip › biology-3864442-supplementary/Figure S1.png]
